# Supplementary material for: Experimental guidance for discovering genetic networks through hypothesis reduction on time series
Source: PLoS Comput Biol. 2022 Oct 10;18(10):e1010145. doi: 10.1371/journal.pcbi.1010145 (PMC9584434; doi:10.1371/journal.pcbi.1010145)
Supplement: S1 Table — (PDF) [file pcbi.1010145.s001.pdf]

| Edge Finding for the Synthetic Network | | | | |
|---|---|---|---|---|
| Dataset | # edges analyzed | # true positives pld $\geq 0.98$ | # false positives pld $\geq 0.98$ | # true positives (out of 10) in top-ranked LEM edges |
| Fig 3B | 98 | $4.60 \pm 0.49$ | $0.00 \pm 0.00$ | $9.80 \pm 0.40$ |
| Fig 3C | 98 | $4.80 \pm 0.40$ | $0.00 \pm 0.00$ | $8.80 \pm 0.40$ |
| Fig 3D | 98 | $2.60 \pm 0.80$ | $0.00 \pm 0.00$ | $9.00 \pm 0.00$ |

**Table S1. Synthetic Network Table of Results for Edge Finding.** All numbers are means over five separate runs of the Inherent Dynamics Pipeline plus/minus one standard deviation. Column 1 contains the dataset that was analyzed (see Fig 3 in the main text). Column 2 reports the the total number of pairwise interactions modeled by LEM; in this case that is $7^2$ interactions between 7 nodes multiplied by 2 for positive and negative regulation. Columns 3 and 4 report the number of true positive and false positive edges with a LEM probability score greater than the chosen threshold. This indicates the number of ground truth and non-ground truth edges in the seed network for the network finding step out of 10 total ground truth edges (see Fig 3A in the main text). Column 5 is the number of ground truth edges available for network sampling in the network finding step; i.e. the number of ground truth edges out of 10 that are in the top-ranked LEM edges, which has approximately 45 edges. It varies slightly according to the size of the seed network.
