# Supplementary material for: Experimental guidance for discovering genetic networks through hypothesis reduction on time series
Source: PLoS Comput Biol. 2022 Oct 10;18(10):e1010145. doi: 10.1371/journal.pcbi.1010145 (PMC9584434; doi:10.1371/journal.pcbi.1010145)
Supplement: S2 Table — (PDF) [file pcbi.1010145.s002.pdf]

Network Finding for the Synthetic Network				
Dataset	# consistent networks (out of 2000)	# top networks	# false negatives	# true negatives correctly identified
Fig 3B	873 ± 221	49 ± 20	0.00 ± 0.00	24.20 ± 1.33
Fig 3C	1043 ± 102	30 ± 2	0.00 ± 0.00	26.00 ± 0.89
Fig 3D	1075 ± 183	83 ± 51	0.20 ± 0.40	20.20 ± 2.79

Table S2. Synthetic Network Table of Results for Network Finding. All numbers are means over five separate runs of the Inherent Dynamics Pipeline plus/minus one standard deviation. Column 1: The dataset that was analyzed (see Fig 3 in the main text). Column 2: The number of sampled networks that have at least one pattern match for at least one dataset out of 2000 sampled networks. Column 3: Top networks are those networks with an oscillation score of 100% and a pattern match score of 50% or above. Column 4: The number of true positive edges with a zero edge prevalence score, i.e., those that are false negatives. These are above and beyond those missing from local edge ranking, as indicated in the last column of S1 Table. Column 5: The number of false positives that are correctly identified, out of approximately 45.
