# Supplementary material for: Experimental guidance for discovering genetic networks through hypothesis reduction on time series
Source: PLoS Comput Biol. 2022 Oct 10;18(10):e1010145. doi: 10.1371/journal.pcbi.1010145 (PMC9584434; doi:10.1371/journal.pcbi.1010145)
Supplement: S3 Table — (PDF) [file pcbi.1010145.s003.pdf]

Edge	Mean edge prevalence score ± 1 standard deviation	Median global edge ranking	Median local edge ranking
B=act(C)	100.0% ± 0.0%	1	1
C=rep(D)	77.0% ± 28.2%	2	2
F=rep(B)	100.0% ± 0.0%	3	3
E=act(F)	100.0% ± 0.0%	4	4
A=act(F)	87.9% ± 24.3%	5	5
D=rep(E)	58.6% ± 27.4%	7	13
C=act(E)	46.2% ± 19.7%	7	25
E=act(A)	43.1% ± 9.5%	8	30
C=act(A)	37.2% ± 12.4%	9	26
A=rep(B)	14.8% ± 4.2%	12	22
E=rep(B)	13.6% ± 2.9%	13	23
A=rep(C)	7.2% ± 2.1%	16	28
E=rep(C)	7.7% ± 2.4%	17	36
C=act(F)	4.5% ± 3.2%	19	33
A=rep(D)	4.2% ± 1.9%	20	35
F=rep(C)	2.9% ± 5.7%	44	25
F=rep(E)	1.4% ± 2.9%	44	32
G=rep(A)	0.0% ± 0.0%	45	8
G=act(G)	0.0% ± 0.0%	45	10
G=act(E)	0.0% ± 0.0%	45	11
G=rep(D)	0.0% ± 0.0%	45	11
G=act(F)	0.0% ± 0.0%	45	12
G=rep(E)	0.0% ± 0.0%	45	13
G=act(A)	0.0% ± 0.0%	45	13
G=rep(F)	0.0% ± 0.0%	45	14
G=act(D)	0.0% ± 0.0%	45	15
G=rep(C)	0.0% ± 0.0%	45	16
G=act(B)	0.0% ± 0.0%	45	19
G=act(C)	0.0% ± 0.0%	45	21
G=rep(B)	0.0% ± 0.0%	45	24
F=rep(A)	0.0% ± 0.0%	45	35
F=rep(D)	0.0% ± 0.0%	45	45

Table S3. Median edge rankings and average edge prevalence scores over five computations for Fig 3B in the main text. These are the edges present in the top-ranked LEM edges in all five computations. The notation A=act(B) should be read "A activated by B". Boxed global edge ranks denote ground truth edges. Notice that the ground truth edge D repressed by A was not a top-ranked LEM edge for at least one computation and is therefore not listed. All edges with a zero prevalence score are given the worst possible rank. The edges are sorted by median global edge ranking.
