# Supplementary material for: Experimental guidance for discovering genetic networks through hypothesis reduction on time series
Source: PLoS Comput Biol. 2022 Oct 10;18(10):e1010145. doi: 10.1371/journal.pcbi.1010145 (PMC9584434; doi:10.1371/journal.pcbi.1010145)
Supplement: S4 Table — (PDF) [file pcbi.1010145.s004.pdf]

Edge	Mean edge prevalence score ± 1 standard deviation	Median global edge ranking	Median local edge ranking
F=rep(B)	100.0% ± 0.0%	1	1
E=rep(B)	100.0% ± 0.0%	2	2
B=act(C)	100.0% ± 0.0%	3	3
D=rep(A)	88.7% ± 22.7%	4	4
C=rep(D)	100.0% ± 0.0%	5	5
A=act(E)	63.3% ± 14.5%	6	14
A=act(F)	48.6% ± 8.0%	7	6
D=rep(F)	38.1% ± 6.4%	8	22
F=act(E)	33.1% ± 3.0%	9	36
C=act(F)	27.4% ± 5.7%	10	31
C=act(E)	18.5% ± 2.8%	12	33
C=act(A)	14.5% ± 2.2%	13	20
F=act(D)	10.5% ± 2.2%	15	32
F=rep(A)	5.7% ± 3.6%	17	36
E=act(D)	4.7% ± 1.9%	17	30
F=rep(C)	2.5% ± 2.4%	19	27
D=act(B)	0.6% ± 1.3%	45	34
G=act(A)	0.0% ± 0.0%	45	7
G=act(G)	0.0% ± 0.0%	45	10
G=rep(A)	0.0% ± 0.0%	45	10
G=act(E)	0.0% ± 0.0%	45	11
G=rep(B)	0.0% ± 0.0%	45	11
G=act(F)	0.0% ± 0.0%	45	13
G=rep(C)	0.0% ± 0.0%	45	15
G=act(C)	0.0% ± 0.0%	45	15
G=act(B)	0.0% ± 0.0%	45	16
G=rep(E)	0.0% ± 0.0%	45	16
G=rep(D)	0.0% ± 0.0%	45	17
G=rep(F)	0.0% ± 0.0%	45	18
G=act(D)	0.0% ± 0.0%	45	22
B=rep(D)	0.0% ± 0.0%	45	24
E=rep(C)	0.0% ± 0.0%	45	26
B=act(A)	0.0% ± 0.0%	45	28
A=rep(B)	0.0% ± 0.0%	45	29
F=act(F)	0.0% ± 0.0%	45	37
B=act(F)	0.0% ± 0.0%	45	41
F=rep(E)	0.0% ± 0.0%	45	41
F=act(G)	0.0% ± 0.0%	45	43

Table S4. Median edge rankings and average edge prevalence scores over five computations for Fig 3C in the main text. These are the edges present in the top-ranked LEM edges in all five computations. The notation A=act(B) should be read "A activated by B". Boxed global edge ranks denote ground truth edges. Notice that the ground truth edges D repressed by E and E activated by F were not top-ranked LEM edges for at least one computation and are therefore not listed. All edges with a zero edge prevalence score are given the worst possible rank. The edges are sorted by median global edge ranking.
