# Supplementary material for: Experimental guidance for discovering genetic networks through hypothesis reduction on time series
Source: PLoS Comput Biol. 2022 Oct 10;18(10):e1010145. doi: 10.1371/journal.pcbi.1010145 (PMC9584434; doi:10.1371/journal.pcbi.1010145)
Supplement: S5 Table — (PDF) [file pcbi.1010145.s005.pdf]

Edge	Mean edge prevalence score ± 1 standard deviation	Median global edge ranking	Median local edge ranking
A=act(F)	100.0% ± 0.0%	1	1
F=rep(B)	100.0% ± 0.0%	2	2
B=rep(D)	52.9% ± 18.0%	3	25
C=act(A)	60.6% ± 4.1%	4	19
B=act(C)	58.5% ± 19.1%	5	4
D=rep(A)	40.3% ± 8.9%	6	27
B=act(A)	33.5% ± 16.8%	7	17
E=act(F)	42.0% ± 26.1%	9	10
C=act(F)	18.1% ± 5.2%	11	28
D=rep(F)	12.8% ± 6.5%	12	9
F=act(D)	12.2% ± 7.0%	13	30
A=act(E)	12.7% ± 5.8%	14	29
F=rep(C)	9.6% ± 6.3%	14	21
F=rep(A)	16.9% ± 15.4%	15	32
C=act(E)	44.3% ± 45.5%	17	3
A=rep(B)	7.9% ± 2.5%	17	33
E=rep(B)	25.0% ± 37.6%	18	5
D=rep(E)	4.9% ± 2.5%	19	6
B=act(E)	8.5% ± 7.7%	20	34
E=rep(C)	3.5% ± 5.1%	22	34
E=act(D)	0.8% ± 0.9%	23	36
F=rep(D)	0.3% ± 0.4%	42	38
G=act(D)	0.0% ± 0.0%	42	7
G=rep(C)	0.0% ± 0.0%	42	8
G=rep(B)	0.0% ± 0.0%	42	11
G=act(G)	0.0% ± 0.0%	42	12
G=act(F)	0.0% ± 0.0%	42	14
G=act(E)	0.0% ± 0.0%	42	14
G=act(A)	0.0% ± 0.0%	42	16
G=rep(D)	0.0% ± 0.0%	42	19
G=act(C)	0.0% ± 0.0%	42	19
G=rep(A)	0.0% ± 0.0%	42	21
G=rep(E)	0.0% ± 0.0%	42	21
G=rep(F)	0.0% ± 0.0%	42	22
G=act(B)	0.0% ± 0.0%	42	24
E=act(E)	0.0% ± 0.0%	42	37
F=act(F)	0.0% ± 0.0%	42	39

Table S5. Median edge rankings and average edge prevalence scores over five computations for Fig 3D in the main text. These are the edges present in the top-ranked LEM edges in all five computations. The notation A=act(B) should be read “A activated by B”. Boxed global edge ranks denote ground truth edges. Notice that the ground truth edge C repressed by D was not a top-ranked LEM edge for at least one computation and is therefore not listed. All edges with a zero edge prevalence score are given the worst possible rank. The edges are sorted by median global edge ranking.
