# Supplementary material for: Experimental guidance for discovering genetic networks through hypothesis reduction on time series
Source: PLoS Comput Biol. 2022 Oct 10;18(10):e1010145. doi: 10.1371/journal.pcbi.1010145 (PMC9584434; doi:10.1371/journal.pcbi.1010145)
Supplement: S6 Table — (PDF) [file pcbi.1010145.s006.pdf]

| Edge Finding for Yeast Cell Cycle | | | | |
|---|---|---|---|---|
| Scenario | # edges analyzed | # subst. edges pld $\geq 0.98$ | # unsubst. edges pld $\geq 0.98$ | # subst. edges (out of 24) in top-ranked LEM edges |
| $S^+A^+$ | 108 | $4.2 \pm 0.75$ | $1.40 \pm 0.80$ | 22 $\pm$0.0 |
| $S^+A^-$ | 162 | $2.80 \pm 0.40$ | $0.00 \pm 0.00$ | $18.40 \pm 1.02$ |
| $S^-A^+$ | 176 | $3.60 \pm 0.80$ | $2.00 \pm 1.55$ | 14.6 $\pm$1.36 |
| $S^-A^-$ | 242 | $2.20 \pm 0.40$ | $0.40 \pm 0.80$ | $14.00 \pm 0.00$ |

**Table S6. Yeast Cell Cycle Table of Results for Edge Finding.** All numbers are means over five separate runs of the Inherent Dynamics Pipeline plus/minus one standard deviation. Column 1: The decreasing information available to an Inherent Dynamics Pipeline run. Column 2 reports the number of edges modeled by LEM, which is dependent on the presence of annotations (decreases the number of edges) and unsubstantiated nodes (increases the number of edges). Columns 3 and 4 report the number of substantiated and unsubstantiated edges with a LEM probability score greater than the chosen threshold; i.e. the edges in the seed network for the network finding step. Column 5 is the number of substantiated edges available to the network sampling in the network finding step out of 24 (see Table 6 in the main text). There are approximately 80 top-ranked LEM edges for each of the five computations. It varies slightly according to the size of the seed network.
