# Supplementary material for: Experimental guidance for discovering genetic networks through hypothesis reduction on time series
Source: PLoS Comput Biol. 2022 Oct 10;18(10):e1010145. doi: 10.1371/journal.pcbi.1010145 (PMC9584434; doi:10.1371/journal.pcbi.1010145)
Supplement: S7 Table — (PDF) [file pcbi.1010145.s007.pdf]

Network Finding for Yeast Cell Cycle				
Scenario	# consistent networks (out of 4000)	# top networks	# subst. edges with 0 prev. score	# unsubst. edges with 0 prev. score
S^+A^+	1678 ±281	165 ±92	6.0 ± 1.4	15.8 ± 5.2
S^+A^-	2436 ± 274	285 ± 109	2.80 ± 0.98	10.20 ± 4.87
S^-A^+	2452 ±140	649 ± 95	2.00 ± 1.41	13.00 ± 2.61
S^-A^-	2495 ± 303	447 ± 234	2.60 ± 1.36	14.80 ± 4.07

Table S7. Yeast Cell Cycle Table of Results for Network Finding. All numbers are means over five separate runs of the Inherent Dynamics Pipeline plus/minus one standard deviation. Column 1: The decreasing amounts of information available to an Inherent Dynamics Pipeline run. Column 2: The number of sampled networks that have at least one pattern match for at least one dataset out of 4000 sampled networks. Column 3: Top networks are those networks with an oscillation score of 10-40% and a pattern match score of 100%. Column 4: The number of substantiated edges with a zero edge prevalence score, meaning they are probable false negatives. Column 5: The number of unsubstantiated edges with a zero edge prevalence score, i.e. probable true negatives. For example, in row 1, six substantiated edges are probable false negatives on average during the network finding step above and beyond the two substantiated edges lost from the top-ranking LEM list due to a low edge ranking (see the last column of S6 Table in row 1 showing 22/24 edges in the top-ranked LEM edges). In addition, on average 16 unsubstantiated edges are identified as probable true negatives (last column).
