# Supplementary material for: Experimental guidance for discovering genetic networks through hypothesis reduction on time series
Source: PLoS Comput Biol. 2022 Oct 10;18(10):e1010145. doi: 10.1371/journal.pcbi.1010145 (PMC9584434; doi:10.1371/journal.pcbi.1010145)
Supplement: S1 File — (PDF) [file pcbi.1010145.s008.pdf]

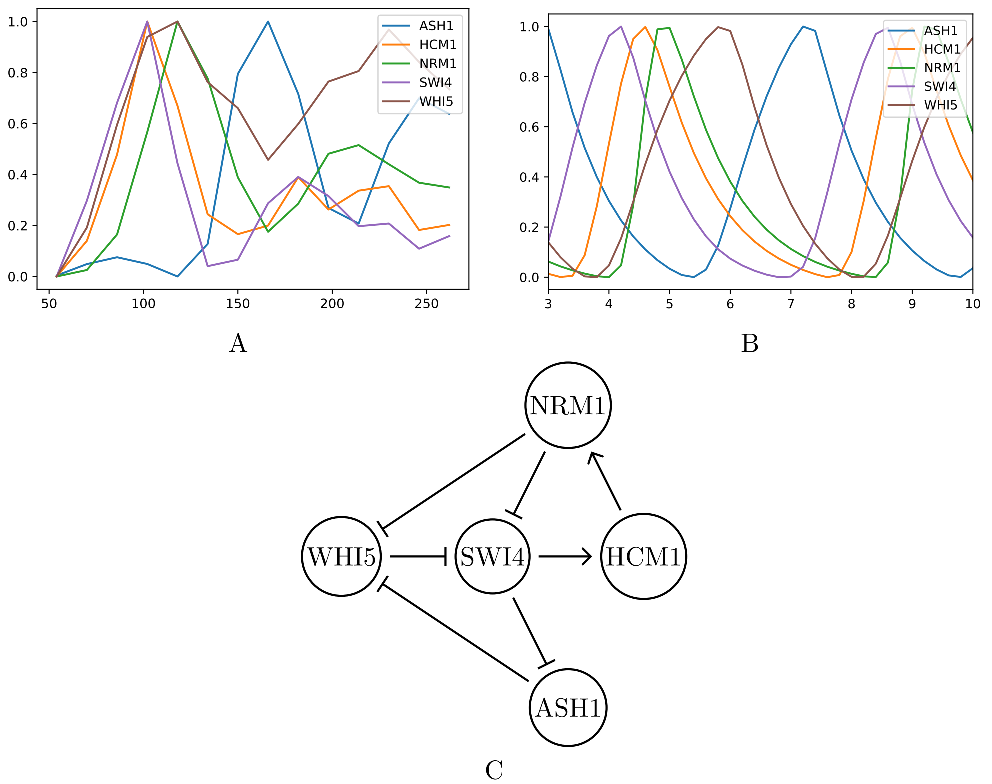

Fig 1. A. Replicate 2 of the wild-type microarray data from [1]. This is a subset of one of the two datasets used in Section 2.2.2 in the main text. B. Hill model simulations of a top network (shown in C) chosen from one replicate of the $S^{-}A^{-}$ scenario. Decay, production, and threshold parameters were randomly chosen from a DSGRN parameter region that showed stable oscillations. The Hill coefficient was set to 8 and each time trace was required to exhibit a fold change of at least 3. The first set of parameters that fulfilled the fold-change condition and pattern-matched the network model was accepted and parameter sampling ceased for that DSGRN parameter region. No effort was made to match the maximum amplitude, amplitude decay (due to gradual loss of cell synchrony during the experiment), or period of the data, as these are not features of the time series used in pattern matching. Therefore the data are normalized, the time axis is not aligned, and the simulations do not show damped oscillations. Notice however that the maxima and minima of the dataset and the simulation occur in a consistent order, particularly the sequence of minima starting at ASH1. The network topology in C was the second network chosen randomly from the list of top networks, as we could not find a match to the data when simulating the first network at a reasonable Hill coefficient.

Hill model equations for the network in Fig 1C. Let A, H, N, S, and W represent ASH1, HCM1, NRM1, SWI4, and WHI5, respectively. Subscript order on the parameters follows the DSGRN convention, so that parameter $p_{b,a}$ is associated to the edge $a \to b$.

$$
\begin{aligned}
\dot{A} &= -A + L_{A,S} + (U_{A,S} - L_{A,S})\frac{\theta_{A,S}^n}{\theta_{A,S}^n + S^n} \\
\dot{H} &= -H + L_{H,S} + (U_{H,S} - L_{H,S})\frac{S^n}{\theta_{H,S}^n + S^n} \\
\dot{N} &= -N + L_{N,H} + (U_{N,H} - L_{N,H})\frac{H^n}{\theta_{N,H}^n + H^n} \\
\dot{S} &= -S + \left(L_{S,N} + (U_{S,N} - L_{S,N})\frac{\theta_{S,N}^n}{\theta_{S,N}^n + N^n}\right)\left(L_{S,W} + (U_{S,W} - L_{S,W})\frac{\theta_{S,W}^n}{\theta_{S,W}^n + W^n}\right) \\
\dot{W} &= -W + \left(L_{W,N} + (U_{W,N} - L_{W,N})\frac{\theta_{W,N}^n}{\theta_{W,N}^n + N^n}\right)\left(L_{W,A} + (U_{W,A} - L_{W,A})\frac{\theta_{W,A}^n}{\theta_{W,A}^n + A^n}\right)
\end{aligned}
\tag{1}
$$

Parameter	Value
$L_{A,S}$	0.26071767470976087
$U_{A,S}$	2.517100290924193
$\theta_{A,S}$	0.6587685127843289
$L_{H,S}$	0.14597324539008544
$U_{H,S}$	2.2390018718738096
$\theta_{H,S}$	1.8876827627001234
$L_{N,H}$	0.06471121142183141
$U_{N,H}$	3.9295649073789116
$\theta_{N,H}$	1.3207172292722547
$L_{S,N}$	0.9435366657472505
$U_{S,N}$	2.843230252516142
$\theta_{S,N}$	0.23874104800489743
$L_{S,W}$	0.0999753501363841
$U_{S,W}$	1.2167803455254358
$\theta_{S,W}$	0.1875091204324653
$L_{W,N}$	0.024274867327902308
$U_{W,N}$	0.5119324500033974
$\theta_{W,N}$	1.9864252043090662
$L_{W,A}$	0.04137012824348931
$U_{W,A}$	1.3916708374087823
$\theta_{W,A}$	0.8736496371062356
n	8

Table 1. Parameterization of ODE system (1) that produced the time traces in Fig 1B. This sample is from DSGRN essential parameter index 2.

References

1. Orlando DA, Lin CY, Bernard A, Wang JY, Socolar JE, Iversen ES, et al. Global control of cell-cycle transcription by coupled CDK and network oscillators. Nature. 2008;453(7197):944–7. doi:10.1038/nature06955.
