# Supplementary material for: Experimental guidance for discovering genetic networks through hypothesis reduction on time series
Source: PLoS Comput Biol. 2022 Oct 10;18(10):e1010145. doi: 10.1371/journal.pcbi.1010145 (PMC9584434; doi:10.1371/journal.pcbi.1010145)
Supplement: Attachment — Submitted filename: PCOMPBIOL-D-22-00666.pdf [file pcbi.1010145.s009.pdf]

Overview

Computational approaches for network inference from high-dimensional gene expression data can be a powerful method to provide experimental guidance. However, many current approaches for network inference (i) require prior biological knowledge, (ii) assume linear relationships between features, (iii) do not incorporate dynamic models, and/or (iv) rely on a single model instead of more reliable ensemble-based predictions. Any one of these can pose limitations if trying to predict networks regulating molecular cycles, eg. cell division or circadian.

This manuscript describes a method for inferring networks that function as biological oscillators. The uniqueness of the approach is that it combines multiple validated computational tools for periodic feature detection and network inference with the intent to ‘prune hypotheses’ of oscillator structure. The output is a prioritized list of regulatory elements as a potentially powerful guide for subsequent experimental validation.

The authors do a nice job of evaluating the method’s performance on synthetic and real cell-cycle data. Addressing the comments below would strengthen the manuscript and provide additional context for those in the community who may be interested in using the pipeline for the intended purpose: network inference for non-model organisms lacking other prior data.

Comments

1. How sensitive is the method to sampling frequency? It seems like node & edge finding from data acquired ‘every 1 h for 48 h’ could be very different from ‘every 4 h for 24 h’. Perhaps the authors could simulate via downsampling on the real yeast dataset?

2. And method sensitivity to data type? For example, performance on qPCR data?

3. [Line 546] “Practically speaking, given current algorithms and technology, this limits networks to about 10 nodes and a maximum of 4 or 5 regulators at any given node; however, only one or two nodes may have 4 or 5 regulators.” The key step here is to find core regulatory elements. How applicable is the current pipeline for identifying the set of nodes from the genome-wide time-series transcriptomic data *de novo*? See point below.

4. Yeast. It is clear that the method can recapitulate known network structure when ‘preloaded’ with substantiated nodes and/or edges. What is less clear (perhaps it is there but could be brought out / made more explicit) is how the pipeline performs assuming zero prior knowledge? It seems that even the least informed of the four scenarios in Fig. 5 ($S^- A^-$) still includes all of the substantiated nodes. In other words, it would be very helpful to directly evaluate performance in terms of the value proposition of the overall method: network inference for novel organisms or core oscillators without much or any prior information.

5. [Line 184] “The local and global edge and node rankings together are the primary metrics prioritizing experiments.” Given their importance, the distinction between ‘local’ and ‘global’ could be made clearer in the introductory section 2.1.3 and throughout. There is a considerable amount of jargon that could be replaced with more straightforward descriptive

language. An example schematic, of the kind in Fig 1, would be very helpful for an uninitiated reader to conceptualize the key aspects of the network finding step. These do become clearer as the authors step thru the examples, but are initially rather confusing.

6. [Line 207] Network space in this case is intractably large, so we were forced to use a sampling technique in network finding, rather than an exhaustive technique as in node finding and edge finding. It isn't clear what is meant here. If this synthetic network of six nodes is 'intractably large' wouldn't that be so for every real dataset?

7. Fig 5 Is the improvement in the S^- model mainly driven by correct predictions of two unsubstantiated genes? Can this pipeline be used to extend the knowledge on cell-cycle networks? For example, can it help to identify new genes (nodes) important to the network, beyond the 9 substantiated genes.

8. Figure 4 and Figure 6 legends are confusing. "Nodes located above the red diagonal line indicate an improved global ranking in their node participation score versus their local node rankings." Maybe consistently use 'ranking' instead of 'participation score'?
