# Supplementary material for: Experimental guidance for discovering genetic networks through hypothesis reduction on time series
Source: PLoS Comput Biol. 2022 Oct 10;18(10):e1010145. doi: 10.1371/journal.pcbi.1010145 (PMC9584434; doi:10.1371/journal.pcbi.1010145)
Supplement: Attachment — Submitted filename: ResponseToReferees.pdf [file pcbi.1010145.s010.pdf]

Response to Referees

Experimental Guidance for Discovering Genetic Networks through Hypothesis Reduction on Time Series

by B. Cummins, F.C. Motta, R.C. Moseley, A. Deckard, S. Campione, M. Gameiro, T. Gedeon, K. Mischaikow, S.B. Haase

(submitted to *PLOS Computational Biology*)

We would like to thank the reviewers for their comments and questions that greatly contributed to the improvement of the paper. We provide below a detailed response to each of the issues presented by the referees.

Responses to Referee #1

1. *I don't think the title really matches the content of the manuscript. The term "iterative hypothesis reduction" was used by the authors, but I can't find any iterative experiments conducted in this paper. Given the core nodes, interactions, or networks identified in the first round, I'm curious how the authors might design the next round of experiments. The synthetic network could be used to demonstrate how to further reduce the number of possible networks.*

   Response: This is a good point. We use iterative in the sense that it is used in the name of software, **I**terative **N**etwork **H**ypoth**e**sis **Re**ductio**n** from **T**emporal **Dynamics**, in which hypothesis reduction is repeated over nodes, edges, and networks. However, we do not address the iteration between computation and experiment, although this is indeed the ultimate aim of using the software. We have addressed this issue by removing "iterative" from the title, but have left it in the name of the pipeline.

2. *The authors have implemented a software named DSGRN in the Inherent pipeline. In the original paper [42], only small-scale networks with a maximum of 5 nodes were tested. I would recommend the authors benchmark its performance on larger networks in the paper (even in a SI figure/table).*

   Response: The referee points out an issue of great interest to us, as benchmarking in DSGRN is a tricky proposition. The computational complexity of DSGRN is less dependent on the number of nodes and edges than it is on the arrangement of edges. For example, consider the 3-node network with nodes A, B, and C, where each node is regulated by the other two, and so each node has two in-edges and two out-edges. This network has 64,000 DSGRN parameters. Now replace the edge from B to C by a self-edge on A, causing A to participate in three in-edges and three out-edges. This modified network, despite having the same number of nodes and edges, has 305,424 DSGRN parameters, which results in approximately 4x the number of computations. (This does not necessarily correspond to a 4x increase in computational time, because the amount of time per computation also changes nonlinearly, sometimes increasing and sometimes decreasing.) More dramatically, consider a simple loop of 10

nodes, where each node has one in-edge and one out-edge, for a total of 10 edges. This network has 30 DSGRN parameters, and yet there is a 10-node, 10-edge network that admits the modified 3-node network described above as a subnetwork, showing a difference of 10^4 in number of computations.

This aspect of the performance of DSGRN is rooted in a deep problem in algebraic geometry (addressed in the original paper) that is neither easy to explain nor easy to characterize in a meaningful way for benchmarking. Rather than referring to the number of nodes and/or edges, it is more accurate to say that the number of nodes with a large number of in-edges is, through a nonlinear relationship with number of nodes and edges, the limiting computational factor. For ideas of other network arrangements for which we have done comprehensive analysis, see [7, 8, 13].

The last paragraph of the section on DSGRN pattern matching in Methods (pg 22) attempts to address this issue. Here is the previous text:

"There are limitations to the networks for which DSGRN computations are possible. The main challenge is that the combinatorial growth of the number of DSGRN parameter regions causes computations to become prohibitively expensive with an increasing number of edges in the network. Practically speaking, given current algorithms and technology, this limits networks to about 10 nodes and a maximum of 4 or 5 regulators at any given node; however, only one or two nodes may have 4 or 5 regulators."

We have modified the text to this:

"There are limitations to the networks for which DSGRN computations are possible. The main challenge is that the combinatorial growth of the number of DSGRN parameter regions with the number of regulators into a single node can cause computations to become prohibitively expensive. To illustrate the impact of increasing network complexity, consider a simple loop of 4 nodes, where each node has one in-edge and one out-edge. This network has 12 DSGRN parameters. Now, to one node (say A), add three in-edges, i.e., make each of the remaining three nodes (including A) a regulator of A. This change results in nearly 3 million DSGRN parameters. Practically speaking, some empirical exploration is needed to gauge an acceptable level of network complexity, without exceeding about 4 or 5 regulators at a single node."

3. *I would also recommend the authors pick one or two of the top-ranked DSGRN, run ODE simulations (or even discrete simulations), and show that they are able to reproduce the target dynamic behaviors.*

   Response: This is an excellent idea. We took the list of top networks for one of the yeast simulations in the S^-A^- category and chose two at random. For each of these, we constructed Hill models and ran ODE simulations over a set of random parameter samples from each DSGRN parameter that was predicted to have stable oscillations. For one of these, we were able to immediately locate similar behavior to the yeast dataset using a Hill coefficient of 8, although this network topology mimics much of but is not identical to the network topology in which we have the most confidence [2]. These results have been incorporated at the end of the appendix and referenced in the yeast results section on S^-A^- (pg 15) with the following sentence at the end of the section:

   "Moreover, we show that a top network in the $\mathrm{S^-A^-}$ scenario found by Inherent Dynamics Pipeline is capable of reproducing the data using a standard Hill model of gene interactions (Fig 9), indicating that the dynamically coarse approach taken by DSGRN informs the parameterization of more traditional models that might provide insight into unknown cellular processes."

4. *In figure 1, the rightmost part is confusing. The gray arrows point to the minimum of the blue and green curves, but not the red ones. How is it related to the oscillation score or pattern matching score?*

Response: Thank you for catching this. We have corrected the figure to include an arrow in the red curve as well. Please also see the expanded caption requested by Referee #2 and the additional figure requested by Referee #3.

5. *What does a 100% oscillation score mean (page 9, line 227)? Do the authors imply that the network could oscillate with any parameter set?*

   Response: Roughly, yes. We have added the following text to further explain:

   pg 11: "For an explanation of the meaning of a 100% oscillation score, see Section 4.2.3."

   pg 21: "The oscillation score introduced in Table 2.1 quantifies the amount of stable cycling exhibited in the STGs across all essential DSGRN parameters. It is possible, and indeed reasonably common, for a network to exhibit an oscillation score of 100%, which may appear to imply that all parameterizations of an ODE model of the network should stably cycle. The actual implication is more nuanced. While an oscillation score of 100% does not guarantee that a corresponding Hill model oscillates for all parameter selections, it strongly suggests that an ODE Hill function model with a sufficiently high Hill coefficient and initial condition in the corresponding domain of the phase space will either oscillate or exhibit decaying oscillations. These two outcomes may be difficult to distinguish experimentally."

6. *I am not sure I fully understand Table B.1-B.2 (Table B.1 is not referenced in the main text).*

   Response: Thank you for pointing this out. Tables B.1 and B.2 containing supplemental results for the yeast simulations are analogous to Tables A.1 and A.2 for the synthetic network. Namely, they contain extra information on true/false positives/negatives at different points in the edge and node finding steps. Following our presentation for the synthetic network, we added the following sentence (pg 14)

   "Mean outcomes plus/minus one standard deviation for the five runs are shown in Appendix Tables B.1 and B.2."

7. *I'm curious as to how the authors compare their findings with other algorithms.*

   Response: It is true that many biological "network inference" algorithms exist, and leverage a variety of data including high-throughput gene expression in time series. However, to the best of our knowledge, our goal—to uncover the critical components driving a dynamic phenotype—is not shared by previously proposed methods. Instead, most earlier methods aim to uncover functional interactions across the genome, and measure their success according to classification metrics that measure global success of the method to reveal true edges between genes. This makes comparison to earlier algorithms difficult. That said, the edge finding inference step of our proposed pipeline, LEM, was directly compared to six previously published methods: TD-ARACNE, Inferelator, Banjo, Granger Causality, Hill-DBN and Jump3. LEM on its own makes more detailed predictions than these algorithms, and so to even make these comparisons, the predictive power of LEM had to be weakened. As reported in McGoff, 2016 (`https://doi.org/10.1186/s13059-016-1076-z`), LEM outperformed all these algorithms on the task of correctly ranking known interactions above potential interactions that aren't supported by evidence in real networks and true edges above false edges in synthetic networks. Here we demonstrate that incorporating global dynamic information in the form of matching the ordering of mins and maxes in time series improves upon the results achieved by LEM alone, which is based only on data pairs and local regulatory inferences.

8. *The authors have claimed that "statistics of the top ranked networks be used to provide a prioritization of experimental interventions" (page 17, line 426; page 22, line 601). I don't think experimental*

interventions can be easily conducted at the network level. The authors need to clarify how their pipeline output could be related to a feasible intervention experiment.

Response: We completely agree with the referee that interventions on the network level are impractical. Such interventions must occur at the node level and that is the gist of Figures 4 and 6. The information on a network level is transformed into information on a node level. We have attempted to reiterate and clarify that message by altering the sentence on pg 17:

"We suggest that statistics of the top ranked networks be used to provide a prioritization of experimental interventions at the node level by re-ranking nodes according to their prevalence in dynamical network models consistent with experimental data."

Please also see the response to Referee #2, comment 7, below.

9. *Page 3 line 97, the dominant edges provide predictions of the impact of the interventions is confusing.*

   Response: We agree that the current wording of this sentence is confusing. What we meant to convey is that the prevalence of a node across networks that support the observed dynamics of expression in the data can be used to prioritize candidate nodes for experimental interrogation. We have changed the sentence:

   "The dominant regulatory elements in this set of networks provide a prioritized list of experimental interventions and the dominant edges provide predictions of the impact of the interventions."

   to

   "The prevalence of regulatory elements in the resulting set of networks provides a prioritized list of experimental interventions and the prevalence of various edges provides predictions of the impact of these interventions."

Responses to Referee #2

1. *Something I could not really get a sense off is how much IDP relies on the quality of the datasets used as inputs. Measurements and synchronisation noise has always been a big limitation for identifying true periodic genes in transcriptomics data. I am curious of the authors' opinion on this. Have they tried to increase or decrease measurement noise in their synthetic data? If yes, which part of the pipeline and its predictions was the most affected?*

   Response: We agree that data quality issues, such as measurement uncertainty, sampling rate, and loss of population synchrony over time, will impact the clarity and accuracy of the conclusions we can draw with the IDP. The effects of data quality and noise have been explored previously for each module in the IDP.

   In Deckard, 2013 (`https://doi.org/10.1093/bioinformatics/btt541`) the authors systematically addressed these concerns specifically for periodicity detection algorithms through extensive testing across a variety of synthetic curve shapes and real datasets from multiple organisms under various noise levels, periods, phase shifts and sampling rates. One of the key takeaways from this study is that different algorithms (with their different computational definitions of rhythmicity) have different strengths and weaknesses and different sensitivities to data challenges. In principal, one could substitute any periodicity finding algorithm into step 1 of the IDP, and the quality of the initial node rankings will reflect the quality of that algorithm to detect core nodes given the nature of the data. In Deckard 2013, the authors found that curve shape was the largest determinant of performance degradation

under increased noise and reduced sampling rate since many periodicity detection methods are based on an assumption about the shape of periodic signals.

In Motta, 2022 (https://doi.org/10.1186/s12859-022-04627-9) the authors address the harder question of identifying core transcriptional regulators across a collection of biological organisms with and without transcription factor annotations and find that certain features of dynamic expression are skillful at identifying core nodes even without any annotations, but that often non-transcriptional regulators also exhibit these features, which highlights the importance of accurate prior biological knowledge.

Furthermore, in McGoff, 2016 (https://doi.org/10.1186/s13059-016-1076-z) the authors explored the effect of noise and incomplete prior information on the quality of LEM's local inferences. Interestingly, added noise did not appear to greatly reduce LEM's performance (Additional file 1: Section 6 and Additional file 19). However, incremental improvements were seen with the inclusion of additional prior annotation information, this time in the form of potential node type: a repressor, an activator, or both (Additional file 1: Section 6 and Additional file 15).

Lastly, in [1], a sequence of different noise levels was applied to the time series discretization method on which DSGRN pattern matching is based. There is notable sensitivity to the amount of noise introduced, specifically because the addition of a hypothesized noise level decreases the number of local mins and maxes that participate in DSGRN pattern matching. Ideally, this noise level is chosen from the dataset itself in order to smooth the curve; however, we currently have no automated way of making an intelligent choice.

Although it is hard to quantitate which step is most affected, we suspect that the node finding and network finding steps are most sensitive to noise in the data, due to a strong dependence on curve shape.

We have added the following text to the first paragraph of Section 2.2 Applications:

"The synthetic data do not include any added noise; we remark that the effects of data quality and noise have been explored previously for each module in the Inherent Dynamics Pipeline [5, 11, 9, 1]."

2. *It is unclear to me from the budding yeast analysis presented in this paper how good IDP would be at identifying previously unknown regulatory nodes. Did I miss it? Could the authors comment please?*

   Response: In this paper we did not specifically address this question. However, in both Motta, 2022 (https://doi.org/10.1186/s12859-022-04627-9), and McGoff, 2016 (https://doi.org/10.1186/s13059-016-1076-z), which introduce the algorithms used in IDP step 1 (node finding) and step 2 (edge finding), the authors showed that periodicity detection alone and local regulatory inferences alone could be used to uncover previously unknown core regulatory nodes. Table 2 in Motta, 2022 shows highly ranked candidate nodes for which there is experimental evidence that they are targeted by and target known core nodes in circadian and cell-cycle systems. In McGoff, 2016, LEM was used to prioritize a list of potential core nodes in a circadian system by ranking nodes according to how likely they were to regulate and be regulated by known core elements. 40% of the tested proposed candidates were found to have significant circadian phenotype.

3. *Figure 1: I find it a bit difficult to relate the diagrammes shown on the figure with the node/edges finding process. Maybe an extended legend could help?*

   Response: We agree that the caption is too brief. Here is the expanded version:

   "Schematic of the three stages of the Inherent Dynamics Pipeline in which each step uses different features of the input gene expression time series data. In the node finding step, each gene expression

time trace is used independently to score the strength of periodicity. Genes with stronger periodicity and higher amplitude are hypothesized to be part of the core network and are correspondingly ranked higher. Top ranked nodes are passed into the edge finding step, where time series are used in pairs to score the likelihood of a positive or negative regulatory event in one or the other direction. A ranking is determined using high to low likelihoods and top ranked edges are passed to the network finding step, where subsets of gene expression data consisting of three or more time traces are compared to the global dynamics of network models to produce top ranking networks that are consistent with the order of peaks and troughs across the time series. Statistics of these top ranked networks are used to suggest experimental intervention at the node level."

We also direct the referee to the new Figure 2 focusing on the network finding step requested by Referee #3.

4. *P4 L117: "Experimental approaches to accomplish this identification task are largely intractable.". What do the authors mean by intractable here.*

   Response: We replaced the sentence with the following clarification:

   "Approaches to extract a small number of core genes from tens of thousands would be experimentally time-consuming and expensive, and thus largely intractable."

5. *P5, L125: "top of the list is enriched for core regulatory elements most critical to controlling oscillatory dynamics". I don't understand why regulatory elements are expected to be themselves oscillating? Their activation could be post-transcriptional, which is quite commonly seen in the case of cell-cycle regulation. Could the authors elaborate on this please?*

   Response: It is certainly true that the abundance of regulatory elements need not oscillate, and we are aware that key elements implicated in the core oscillator may be post-transcriptional. One of our findings in [11] was that known core elements of circadian and cell-cycle oscillators were themselves oscillating. Oscillation is just one of the features we discovered that characterize these regulatory elements, and thus they tend to cluster at the top of a rank ordered list based on periodicity measures. Even in the case of the central regulatory kinase for the cell cycle (CDK), a good proxy for CDK activity in budding yeast is the abundance of cyclins which oscillate in multiple different phases of the cycle. Certainly, we may miss discovering regulatory elements that are not oscillatory using this method. Moreover, it is clear that regulator activities are affected by post-transcriptional regulation, so like all simplifying assumptions we lose some accuracy. That said, we have very recently developed a new version of DSGRN that models post-transcriptional regulatory events [4]. However, data matching for both transcriptional and post-transcriptional regulation would require synchronized mixed data types, i.e. measurements of the transcriptome, proteome, and possibly phosphorylome. We intend to upgrade the Inherent Dynamics Pipeline in the future with these new modeling capabilities once the appropriate data type integration has been developed for all modules.

6. *Figure 2. It would be nice to have a visual representation of the results in the main paper not just in the Appendix.*

   Response: We agree that a good visualization would be helpful. However, the synthetic network Appendix is composed of seven dense tables containing what we intended to be supplemental results. We have struggled to represent the key results in easily interpretable visualizations.

7. *P9, L207 and Figure 3: This section is unclear. What are the implications of this identifiability problem for the users?*

Response: The implication for the user is that the discovery of a single "true" network structure is not a realistic goal. We have replaced the last two sentences of the indicated section, now on pg 10.

Previous text:

"The large number of consistent networks is a manifestation of an identifiability problem, wherein many networks of differing topologies were capable of producing the observed transcriptional oscillations. The ability of a network model to reproduce a particular dataset was not rare in the set of networks constructed from high ranking LEM edges."

Revision:

"In other words, the ability of a network model to reproduce a particular dataset was not rare in the set of networks constructed from high ranking LEM edges. The large number of consistent networks is a manifestation of an identifiability problem, wherein many networks of differing topologies were capable of producing the observed transcriptional oscillations. This is due to the inherent flexibility of network structure to produce different dynamics. Since a large number of network structures could explain the observed data, we do not seek to provide a "true" network to the user. Instead, we use statistics over the aggregate of high performing networks to pinpoint genes of interest for experimental intervention (i.e. Fig 5)."

8. *P10, L235:"...due simply to spurious correlations in their transcriptional...". What do the authors mean by spurious?*

   Response: We expanded the phrase to "spurious correlations due to limited precision".

9. *P10, L246: The discussion of condition specific node is interesting. However, I am not too sure how this relates to inference by the ID pipeline. Could the author clarify how their analysis presented on panels e-h affect the use of their pipeline please?*

   Response: We agree that the message was not clear. On pg 12, we made the following clarification.

   Previous text in condition-specific subsection:

   "This presents strong evidence that the five-node subnetwork in Fig 3 (e) can operate as the true core oscillator of the synthetic network under some conditions. This is an example of a case where a strongly connected network is not necessarily a core oscillator and illustrates the crucial role parameters can play in determining the true core."

   New text:

   "This presents strong evidence that the five-node subnetwork in Fig 3 (e) can operate as the true core oscillator of the ground truth network under some parameterizations. We view these different parameterizations as proxies for distinct experimental conditions, such as different growth media or temperature. Thus the Inherent Dynamics Pipeline can distinguish between two strongly connected networks that operate as core oscillators under different cellular conditions. In other words, the Inherent Dynamics Pipeline may not return the same network structures if data are collected under different experimental conditions."

Responses to Referee #3

1. *How sensitive is the method to sampling frequency? It seems like node & edge finding from data acquired 'every 1 h for 48 h' could be very different from 'every 4 h for 24 h'. Perhaps the authors could simulate via downsampling on the real yeast dataset?*

Response: The sensitivities of various steps in the pipeline to data quality, including sampling rates, have been explored in earlier publications (please see the response to Reviewer #2, comment 1, and the references therein). We do not expect that a systematic downsampling of the yeast data in this paper would be informative since we expect that any conclusions about a threshold at which the pipeline performance breaks down (if there is such a threshold) would depend on the particular organism and the network underlying the observed dynamics, and would not generalize. The existence of such a threshold is itself questionable since, for example, the (partial) ordering of mins and maxes in a set of gene expression profiles may depend on which timepoints the curves are (re)sampled and the shapes of the curves.

2. *And method sensitivity to data type? For example, performance on qPCR data?*

   Response: Both DLxJTK and LEM have been used successfully on both microarray and RNAseq data [11, 9] without any measurable difference. DSGRN pattern matching has also been applied to both data types [3, 1]. As long as CT scores from q-PCR analyses could be converted to properly scaled measures of expression (e.g. number of mRNAs) then there is no reason to expect any issues with that data type. That said, because qPCR is measuring expression on a gene-by-gene basis, it is probably not as efficient as high-throughput measurements that allow exploration of the entire genome as needed for inference.

3. *[Line 546] "Practically speaking, given current algorithms and technology, this limits networks to about 10 nodes and a maximum of 4 or 5 regulators at any given node; however, only one or two nodes may have 4 or 5 regulators." The key step here is to find core regulatory elements. How applicable is the current pipeline for identifying the set of nodes from the genome-wide time-series transcriptomic data de novo? See point below.*

   Response: In Motta, 2022 (`https://doi.org/10.1186/s12859-022-04627-9`) it was shown that a combination of dynamic expression features can be used to skillfully rank core transcriptional regulatory elements across a variety of biological organisms and data modalities. As mentioned in response to Referee #2, comment 2, while these rankings contained false positives even at stringent cutoffs, in some cases these false positives may represent discoveries since in there is evidence that the nodes target and are targeted by known core nodes (Table 2 in Motta, 2022). The authors also show the importance of accurate annotation of function for this task: While the dynamic features studied remain skillful at ranking core nodes above non-core without any prior biological knowledge, the sheer volume of potential nodes leads to many false positives which may pose a computational challenge to downstream steps of the IDP to refine the rankings of core nodes.

4. *Yeast. It is clear that the method can recapitulate known network structure when 'preloaded'. with substantiated nodes and/or edges. What is less clear (perhaps it is there but could be brought out / made more explicit) is how the pipeline performs assuming zero prior knowledge? It seems that even the least informed of the four scenarios in Fig. 5 (S^-A^-) still includes all of the substantiated nodes. In other words, it would be very helpful to directly evaluate performance in terms of the value proposition of the overall method: network inference for novel organisms or core oscillators without much or any prior information.*

   Response: We completely agree that it would be useful to evaluate the end-to-end IDP capability on a non-model organism without substantial prior knowledge. However, assessing the performance of the IDP end-to-end is difficult given that mistakes in the early node-finding steps will be propagated into the later steps. The accuracy of the node-finding component of the IDP was explored in detail in a

previous publication [11]. The goal of the analysis in Fig. 5 was to measure the accuracy of combining the local and global inference methods (LEM and DSGRN). To get the best measure, we took the highest confidence nodes, most of which occurred near the top of the DLxJTK ranked list. Confidence was assessed by experimental evidence as documented in the Yeastract database [10, 12]. We identified two nodes near the top of the DLxJTK ranked list that had no corroborating experimental evidence and are therefore low confidence nodes that are likely to be false positives. By "pre-loading" the list with high-confidence nodes and presumed false positives, we had the best chance of evaluating the accuracy of the local and global inference steps. It's worth noting that there was no "pre-loading" of edges.

Certainly, we intend to explore data from non-model organisms. However, a measure of end-to-end accuracy will require experimental validation, and that will likely take some time. We also recognize the importance of the node-finding step in terms of error propagation and are working on additional methods to increase the accuracy of that step.

To the discussion, we added the following statement:

"The performance of the node finding step is well-documented in [11]; in this manuscript, we *a priori* identify groups of high confidence vs low confidence nodes in order to examine the performance of the combination of edge and network finding steps of the Inherent Dynamics Pipeline."

5. *[Line 184] "The local and global edge and node rankings together are the primary metrics prioritizing experiments." Given their importance, the distinction between 'local' and 'global' could be made clearer in the introductory section 2.1.3 and throughout. There is a considerable amount of jargon that could be replaced with more straightforward descriptive language.*

   Response: We appreciate the referee pointing out the confusing language. We provided the following definitions in the introductory material.

   The following text was added to Section 2.1.2: "The word "local" is chosen here to refer to an inference based on data for a single target and regulator, in contrast to a "global" inference performed over all the edges of a purported GRN simultaneously."

   The following text was added to Section 2.1.3: "The term "global" here refers to the ability of a GRN model to holistically reproduce the proper dynamics of a collection of time series."

   While editing the current manuscript we have attempted to remove or clarify confusing jargon when possible, e.g. synthetic vs ground truth network.

6. *An example schematic, of the kind in Fig 1, would be very helpful for an uninitiated reader to conceptualize the key aspects of the network finding step. These do become clearer as the authors step thru the examples, but are initially rather confusing.*

   Response: This is an excellent suggestion. The manuscript now includes Figure 2 that concentrates on the workflow of the network finding step, and is referenced in Section 2.1.3.

7. *[Line 207] Network space in this case is intractably large, so we were forced to use a sampling technique in network finding, rather than an exhaustive technique as in node finding and edge finding. It isn't clear what is meant here. If this synthetic network of six nodes is 'intractably large' wouldn't that be so for every real dataset?*

   Response: The referee is correct; the space of networks is intractably large for anything 3 nodes or larger (given the combinatorics in node identity). This is simply a fact that any holistic network inference tool must face. We clarified with the following text:

Added to Section 2.1.3: "The task of network finding is complicated by the enormity of network space, which precludes exhaustive evaluation of all network models (see Section 4.2.3)."

And to Section 4.2.3: "One further limitation of the network finding step is the size of network space, which precludes exhaustive evaluation and requires sparse sampling. This is because the number of potential regulatory network structures becomes intractably large for combinations of about three nodes or more. For example, given 10 possible gene products, there are 120 choices of 3-node combinations, and for each combination, there are over 14,000 possible network structures with different combinations of positive and negative regulatory interactions [6]. This leads to well over a million possibilities for a 3-node core network even when the number of possible core nodes has been dramatically reduced. To appreciate the rate of growth, consider that the analogous case for 4-node networks. There are 210 node combinations with over 42 million possible network structures for each combination, leading to billions of possibilities. For this reason, any network inference technique that evaluates network behavior as a whole, including ours, is required to sample network space, rather than using an exhaustive technique as in node finding and edge finding."

We removed the sentence "Network space in this case is intractably large, so we were forced to use a sampling technique in network finding, rather than an exhaustive technique as in node finding and edge finding." since the new content addresses the issue more clearly.

8. *Fig 5 Is the improvement in the S^- model mainly driven by correct predictions of two unsubstantiated genes? Can this pipeline be used to extend the knowledge on cell-cycle networks? For example, can it help to identify new genes (nodes) important to the network, beyond the 9 substantiated genes.*

   Response: Indeed, there is experimental evidence that components of the IDP have the capacity to uncover previously unknown regulatory elements that are important to the function of a core oscillator. As stated in the response to Referee #2, comment 2, Table 2 in [11] shows highly ranked candidate nodes for which there is experimental evidence that they are targeted by and target known core nodes in circadian and cell-cycle systems. Also, in [9], LEM was used to prioritize a list of potential core nodes in a circadian system by ranking nodes according to how likely they were to regulate and be regulated by known core elements. 40% of the tested proposed candidates were found to have significant circadian phenotype.

9. *Figure 4 and Figure 6 legends are confusing. "Nodes located above the red diagonal line indicate an improved global ranking in their node participation score versus their local node rankings." Maybe consistently use 'ranking' instead of 'participation score'?*

   Response: That is indeed confusing. The participation scores are plotted, so we removed the mention of ranking from the caption altogether:

   "Nodes located above the red diagonal line indicate an improved global node participation score versus their local node participation score."

References

[1] Eric Berry, Bree Cummins, Robert R Nerem, Lauren M Smith, Steven B Haase, and Tomas Gedeon. Using extremal events to characterize noisy time series. Journal of mathematical biology, 80(5):1523–1557, 2020.

[2] Chun-Yi Cho, Francis C. Motta, Christina M. Kelliher, Anastasia Deckard, and Steven B. Haase. Reconciling conflicting models for global control of cell-cycle transcription. Cell Cycle, 16(20):1965–1978, 2017. PMID: 28934013.

[3] B. Cummins, T. Gedeon, S. Harker, and K. Mischaikow. Model rejection and parameter reduction via time series. SIAM Journal on Applied Dynamical Systems, 17(2):1589–1616, 2018.

[4] Bree Cummins, Marcio Gameiro, Tomas Gedeon, Shane Kepley, Konstantin Mischaikow, and Lun Zhang. Extending combinatorial regulatory network modeling to include activity control and decay modulation, 2022.

[5] Anastasia Deckard, Ron C. Anafi, John B. Hogenesch, Steven B. Haase, and John Harer. Design and analysis of large-scale biological rhythm studies: a comparison of algorithms for detecting periodic signals in biological data. Bioinformatics, 29(24):3174–3180, 09 2013.

[6] Marcio Gameiro, Tomas Gedeon, Shane Kepley, and Konstantin Mischaikow. Rational design of complex phenotype via network models. PLOS Computational Biology, 17(7):1–25, 07 2021.

[7] Tomas Gedeon, Bree Cummins, Shaun Harker, and Konstantin Mischaikow. Identifying robust hysteresis in networks. PLOS Computational Biology, 14(4):1–23, 04 2018.

[8] Shane Kepley, Konstantin Mischaikow, and Elena Queirolo. Global analysis of regulatory network dynamics: equilibria and saddle-node bifurcations. arXiv e-prints, page arXiv:2204.13739, April 2022.

[9] Kevin A. McGoff, Xin Guo, Anastasia Deckard, Christina M. Kelliher, Adam R. Leman, Lauren J. Francey, John B. Hogenesch, Steven B. Haase, and John L. Harer. The local edge machine: inference of dynamic models of gene regulation. Genome Biology, 17(1):214, Oct 2016.

[10] Pedro T Monteiro, Jorge Oliveira, Pedro Pais, Miguel Antunes, Margarida Palma, Mafalda Cavalheiro, Mnica Galocha, Cludia P Godinho, Lus C Martins, Nuno Bourbon, Marta N Mota, Ricardo A Ribeiro, Romeu Viana, Isabel S-Correia, and Miguel C Teixeira. YEASTRACT+: a portal for cross-species comparative genomics of transcription regulation in yeasts. Nucleic Acids Research, 48(D1):D642–D649, 10 2019.

[11] Francis C. Motta, Robert C. Moseley, Bree Cummins, Anastasia Deckard, and Steven B. Haase. Conservation of dynamic characteristics of transcriptional regulatory elements in periodic biological processes. BMC Bioinformatics, 23(94), 2022.

[12] Miguel C Teixeira, Pedro T Monteiro, Margarida Palma, Catarina Costa, Cláudia P Godinho, Pedro Pais, Mafalda Cavalheiro, Miguel Antunes, Alexandre Lemos, Tiago Pedreira, et al. Yeastract: an upgraded database for the analysis of transcription regulatory networks in saccharomyces cerevisiae. Nucleic acids research, 46(D1):D348–D353, 2018.

[13] Ying Xin, Bree Cummins, and Tomas Gedeon. Multistability in the epithelial-mesenchymal transition network. BMC BIOINFORMATICS, 21(1), FEB 24 2020.
